# Diagnostic Challenges and Uterus-Conserving Surgical Management Following Systemic Multi-dose Methotrexate for a Left Interstitial Pregnancy

**DOI:** 10.7759/cureus.117096

**Published:** 2026-09-28

**Authors:** Dayanna A Zuluaga, Sohail Khan, Harkanwal Kaur, Charles Haddad

**Affiliations:** 1 Obstetrics and Gynecology, Touro College of Osteopathic Medicine, Middletown, USA; 2 Surgery, Touro College of Osteopathic Medicine, Middletown, USA; 3 Obstetrics and Gynecology, Hackensack University Medical Center Mountainside, Montclair, USA

**Keywords:** b-hcg, cornual ectopic pregnancy, diagnostic laparoscopy, incomplete resolution, interstitial pregnancy, left salpingectomy, leucovorin, methotrexate, pregnancy of unknown location, transvaginal ultrasound

## Abstract

Interstitial ectopic pregnancy is an uncommon form of ectopic pregnancy and can be associated with a high risk of rupture and hemorrhagic death due to its deep myometrial location. Diagnosis can be challenging because transvaginal ultrasonography (TVUS) may fail to initially identify the implantation site until late in gestation, and the presentation can closely mimic that of an early intrauterine pregnancy or spontaneous abortion. We report the case of a 27-year-old gravida 2 para 1 (G2P1) woman who presented to the emergency department (ED) with vaginal spotting and pelvic cramping at an estimated seven weeks gestational age. Serial serum beta-human chorionic gonadotropin (β-hCG) measurements demonstrated an initial suboptimal rise followed by a plateau and subsequent decline (3,954 → 6,136 → 5,863 → 5,028 mIU/mL). Repeat TVUS identified no intrauterine pregnancy and no discrete adnexal mass, constituting a pregnancy of unknown location (PUL). Following stabilization in the Rapid Decision Unit, the patient was discharged with urgent outpatient obstetrics and gynecology (OB/GYN) follow-up. Interval repeat TVUS raised concern for a left interstitial ectopic pregnancy, prompting hospital readmission for diagnostic laparoscopy, which confirmed a 3-4 cm unruptured left interstitial ectopic pregnancy. Given hemodynamic stability and the high vascularity of the interstitial mass, intraoperative conservative management was elected in lieu of immediate wedge resection. Postoperatively, she received multi-dose systemic methotrexate with leucovorin rescue under co-management with maternal-fetal medicine and was discharged in stable condition. Although β-hCG declined to undetectable levels, interval imaging identified a persistent adnexal mass and a newly developed left hydrosalpinx. Given the patient's desire for future fertility, she underwent laparoscopic left salpingectomy with wedge resection of the left uterine cornu as uterus-conserving definitive management. This case illustrates the diagnostic limitations of TVUS and the critical role of serial β-hCG kinetics in PUL, as well as the feasibility and limitations of uterus-conserving medical and surgical management for interstitial ectopic pregnancy, including the need for long-term surveillance and the role of definitive surgery when resolution after methotrexate is incomplete.

## Introduction

An ectopic pregnancy occurs when a fertilized ovum implants outside the endometrial cavity, most commonly in the fallopian tube. Because these sites cannot accommodate a growing pregnancy, an ectopic gestation is not viable. Continued growth leads to rupture of the surrounding tissue and potentially life-threatening intra-abdominal hemorrhage. Ectopic pregnancy accounts for approximately 2.7% of pregnancy-related deaths in the United States and remains the leading cause of first-trimester maternal mortality [1].

Interstitial pregnancy is a rare subtype, accounting for approximately 2%-4% of all ectopic pregnancies [2, 3]. Clinicians often use the term cornual ectopic pregnancy interchangeably with interstitial pregnancy; however, a distinction exists. A cornual pregnancy refers to implantation within the upper and lateral portions of the uterus and may occur in the setting of a uterine anomaly such as a bicornuate uterus. An interstitial pregnancy, by contrast, refers specifically to implantation within the proximal intramural portion of the fallopian tube as it is enveloped by the myometrium, a segment approximately 0.7 mm in width and 1-2 cm in length [2]. Throughout this report, we use the term interstitial pregnancy to reflect the anatomical location confirmed at surgery. Despite its relative rarity, interstitial ectopic pregnancy carries a maternal mortality rate of 2.0%-2.5%, estimated to be six to seven times higher than that of other tubal ectopic pregnancies, owing to its highly vascularized implantation site and its capacity for silent growth until catastrophic rupture [3, 4]. The myometrial location permits greater distensibility than the thin-walled fallopian tube, allowing the pregnancy to grow undetected, typically until seven to 16 weeks of gestation, at which point rupture involving the highly vascularized cornual vessels can produce rapid and catastrophic hemorrhage [5].

Early diagnosis is particularly challenging. Because the interstitial segment lies within the uterine wall, the gestational sac sits eccentrically, adjacent to the endometrial cavity, and may be misidentified as an eccentrically located intrauterine pregnancy on transvaginal ultrasonography (TVUS), particularly before eight weeks of gestation [4, 5]. Patients may also present with nonspecific symptoms such as mild vaginal bleeding or cramping, which closely mimic a threatened or spontaneous abortion. A pregnancy of unknown location (PUL), defined by a positive serum beta-human chorionic gonadotropin (β-hCG) with neither an intrauterine nor an ectopic pregnancy identified on TVUS, is encountered in approximately 8%-31% of women presenting with first-trimester bleeding, and 7%-20% of these will ultimately be found to have an ectopic pregnancy, including interstitial implantations that may evade early sonographic detection [6]. In this setting, serial serum β-hCG kinetics become the primary diagnostic tool, guiding the clinician toward appropriate escalation of care.

We present a case of left interstitial ectopic pregnancy that initially presented as a PUL, highlighting the diagnostic limitations of TVUS, the interpretation of serial β-hCG trends, and the role of a combined surgical and pharmacological approach in uterus-conserving management, including the course of events when methotrexate resolution proves incomplete and definitive surgical management becomes necessary.

## Case presentation

A 27-year-old gravida 2 para 1 (G2P1) woman at an estimated gestational age of approximately seven weeks by last menstrual period presented to the emergency department (ED) reporting worsening vaginal spotting and lower abdominal cramping since the previous day. She denied fever, heavy vaginal bleeding, dizziness, or syncope.

Her obstetric history was notable for one prior vaginal delivery (G2P1). Past surgical history was notable for a remote appendectomy. She had no known medical conditions and no known drug allergies. Her only active medication was a daily prenatal vitamin. Social history was notable for current alcohol use; she denied tobacco and illicit drug use.

She had been evaluated by an outpatient obstetrician 13 days before presentation, at which time TVUS did not visualize a gestational sac, attributed to early gestational age. A serum β-hCG of 3,954 mIU/mL was obtained at that visit. A repeat value five days later was 6,136 mIU/mL, an increase of 55% over five days; however, when normalized to the standard 48-hour interval, this corresponded to an approximate rise of 19%, well below the expected minimum of 53% required for a normally progressing intrauterine pregnancy [4]. A third value, drawn the day before presentation, was 5,863 mIU/mL, representing a plateau followed by decline. On the day of ED presentation, a fourth value was 5,028 mIU/mL (Table 1).

**Table 1:** Serial serum β-hCG values and clinical interpretation β-hCG: beta-human chorionic gonadotropin; IUP: intrauterine pregnancy; TVUS: transvaginal ultrasonography

β-hCG (mIU/mL)	Change from Prior	Interval	Clinical Interpretation
3,954	Baseline	—	Initial draw; no IUP on TVUS
6,136	+55%	5 days	Suboptimal rise when normalized to 48-hour interval; indeterminate
5,863	−4.4%	7 days	Plateau then decline; ectopic pregnancy suspected
5,028	−14.3%	1 day	Continued decline; no IUP on repeat TVUS

On physical examination, the patient was alert and in no acute distress. Her vital signs were as follows: temperature 36.6°C, heart rate 99 bpm, blood pressure 129/84 mmHg, respiratory rate 18, SpO₂ 100% on room air. The abdomen was soft, non-tender, and non-distended, with normoactive bowel sounds. There was no peritoneal tenderness, guarding, or rebound. No adnexal tenderness was elicited.

TVUS performed that evening demonstrated a uterus of normal size (7.8 × 4.2 × 4.6 cm) with a thickened endometrium measuring 1.6 cm. No intrauterine gestational sac, yolk sac, or embryo was identified. The right ovary was 2.9 × 2.3 × 2.8 cm and unremarkable. The left ovary measured 2.7 × 1.9 × 2.1 cm and contained a corpus luteum measuring 2 × 1.4 × 1.7 cm. No adnexal mass was identified, and no free fluid was seen in the pelvis. The radiologic impression read, "No evidence of intrauterine gestation at this time. Possibilities include very early intrauterine pregnancy, recent abortion, or occult ectopic gestation. Recommend correlation with serum β-hCG and sonographic follow-up."

A complete blood count demonstrated a hemoglobin of 14.4 g/dL, a hematocrit of 42.1%, and a platelet count of 272 × 10³/µL, consistent with hemodynamic stability. The WBC was 10.1 × 10³/µL. A comprehensive metabolic panel was within normal limits with the exception of a mildly elevated alanine aminotransferase (ALT) of 72 U/L. Blood type was O positive; antibody screen was negative. Urinalysis was sent, and a culture was reflex ordered.

The patient was placed in the ED for observation and serial reassessment. She received one liter of intravenous normal saline. The obstetrics and gynecology (OB/GYN) service was consulted; ectopic pregnancy was identified as the leading diagnosis. The patient remained hemodynamically stable throughout the observation period and was discharged the same day with urgent outpatient OB/GYN follow-up arranged, with explicit return precautions for worsening pain, increased vaginal bleeding, or hemodynamic symptoms.

On interval outpatient evaluation, repeat TVUS raised concern for a left interstitial ectopic pregnancy (Figures 1-3). The patient was admitted to the gynecology service one week after her initial presentation. The admitting history and physical documented a 27-year-old woman at an estimated seven weeks gestational age, presenting for evaluation of a suspected ectopic pregnancy with slowly declining β-hCG levels and mild, intermittent vaginal bleeding. She was hemodynamically stable with a soft, non-tender abdomen and no adnexal tenderness.

**Figure 1:**
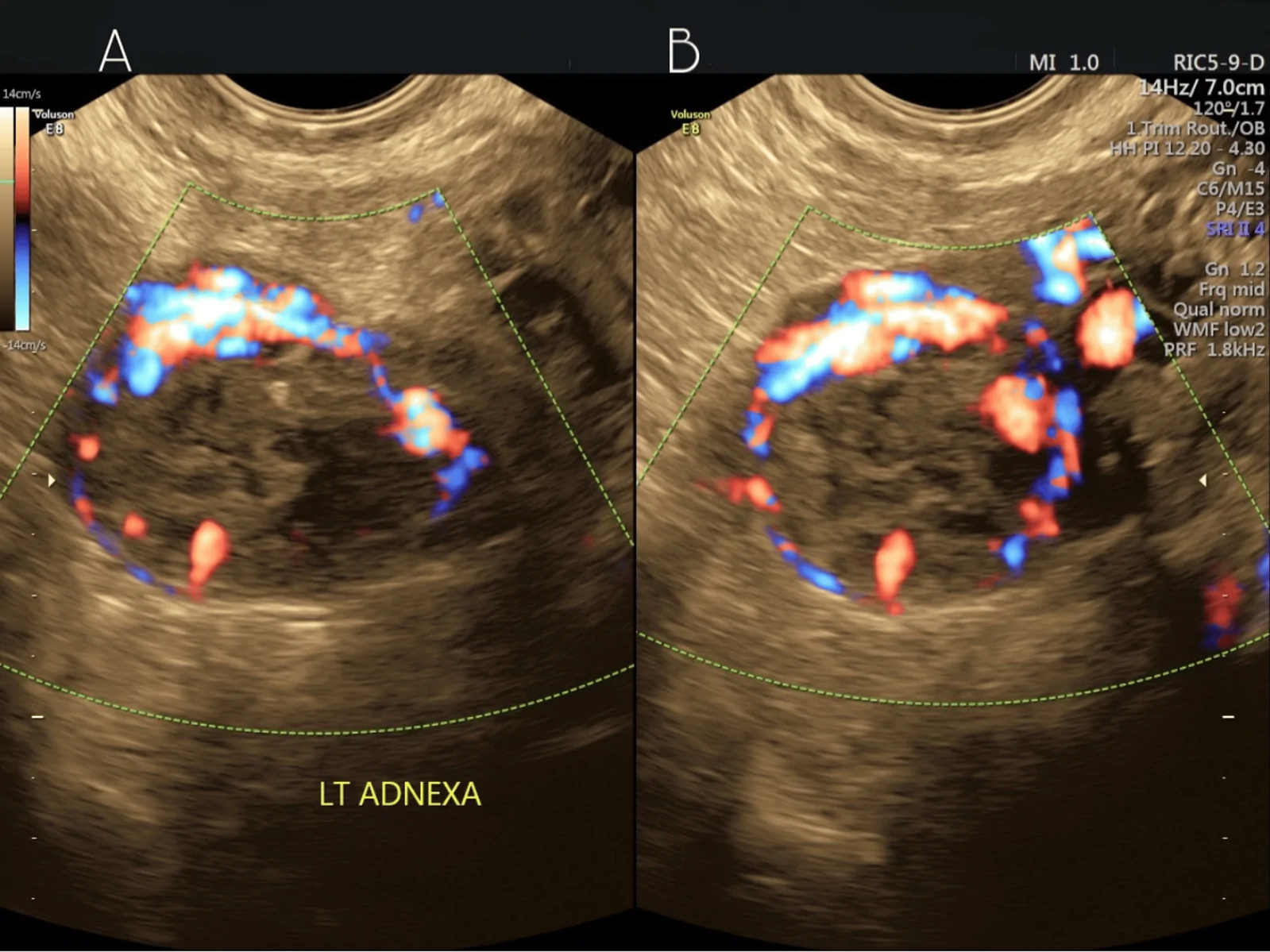
Transvaginal ultrasonography with color Doppler of the left adnexal/interstitial region. (A) Transverse and (B) orthogonal views demonstrate a 2.80 × 2.40 × 2.82 cm heterogeneous mass (arrows) with circumferential peripheral vascularity (arrowheads), consistent with a "ring of fire" pattern. Calipers (1–3) indicate mass dimensions.

**Figure 2:**
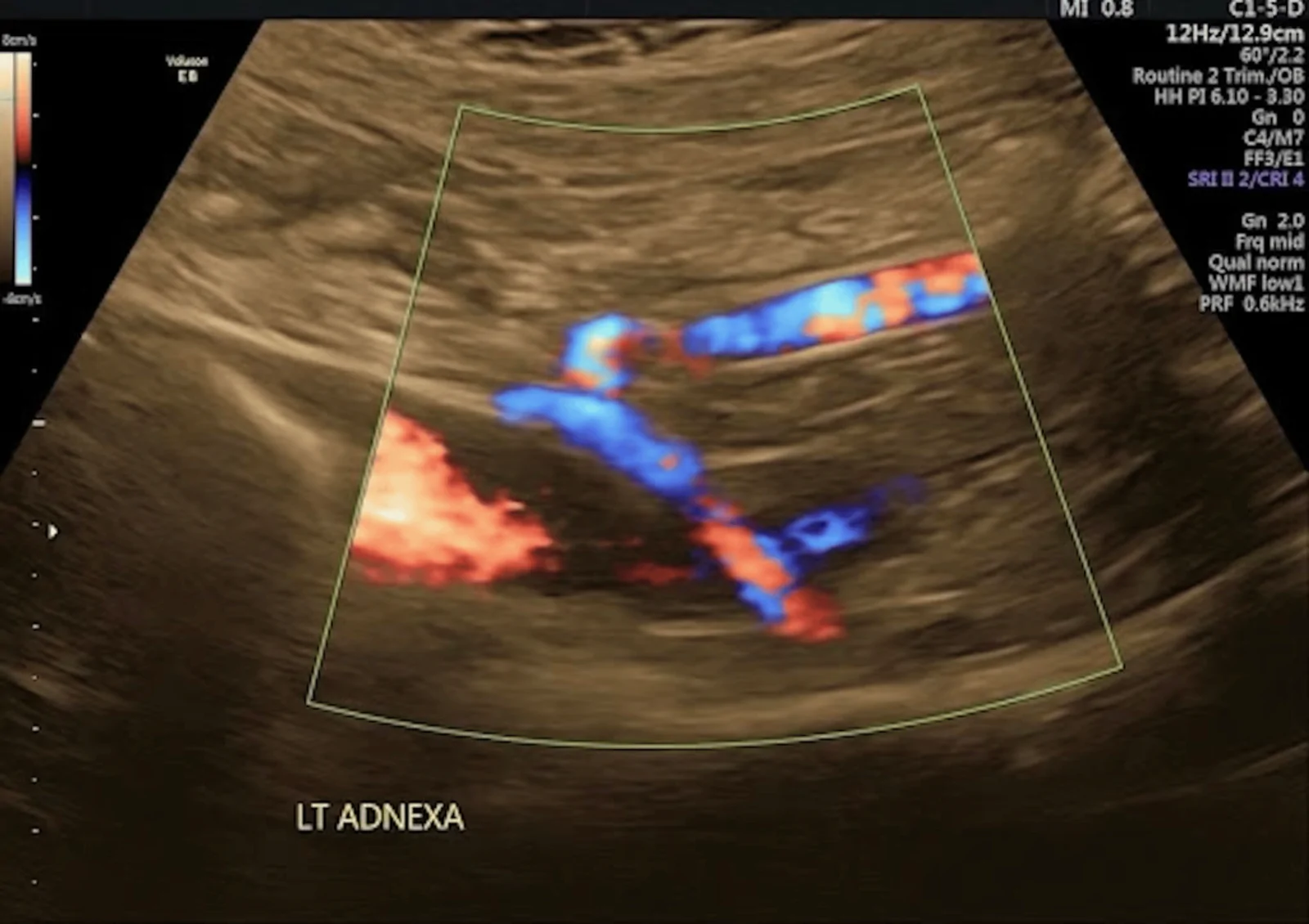
Close-up color Doppler imaging of the left adnexal mass demonstrating prominent and tortuous perilesional vascularity, reflecting the highly vascularized implantation site characteristic of interstitial ectopic pregnancy and underscoring the risk of catastrophic hemorrhage in the event of rupture.

**Figure 3:**
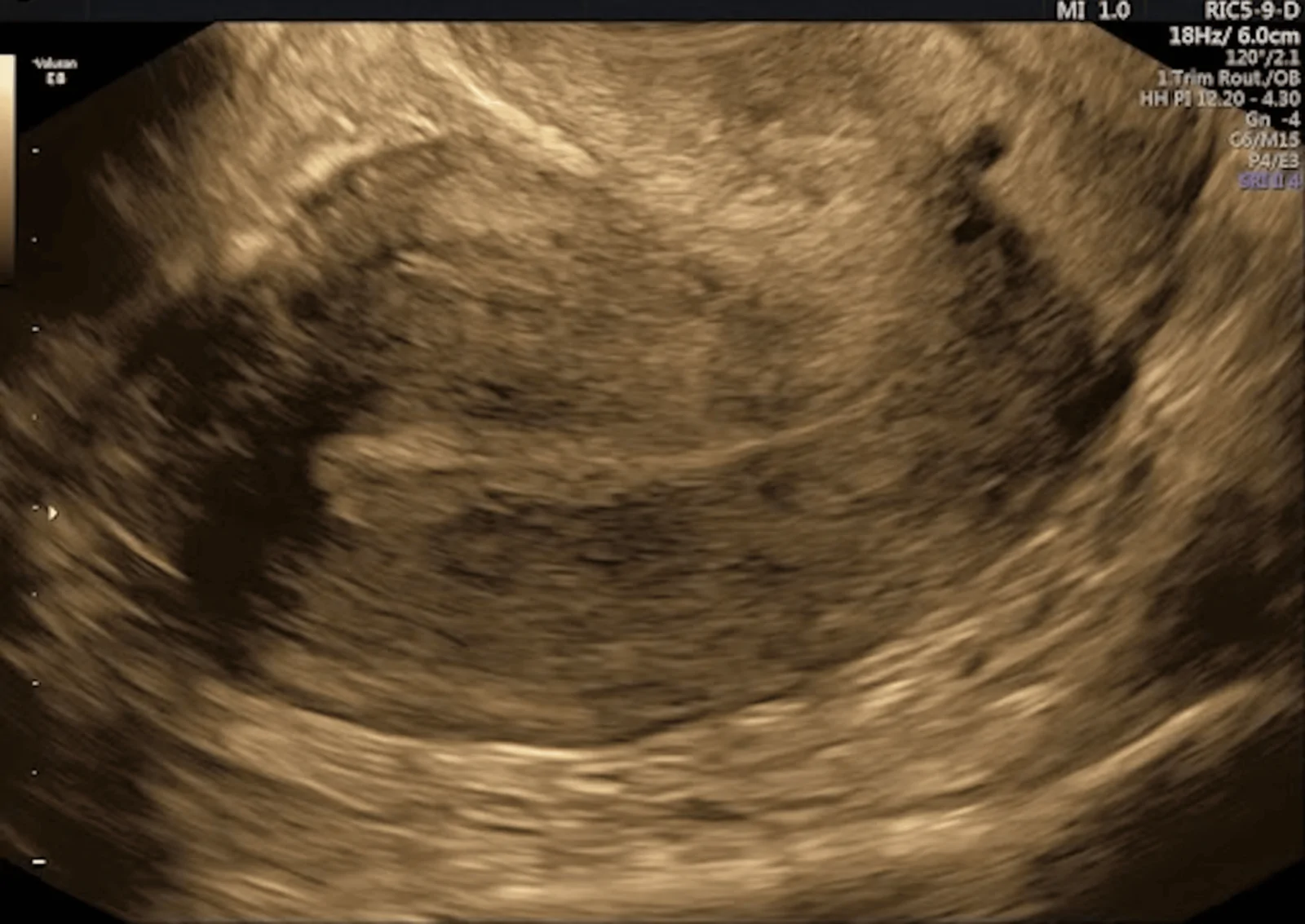
Transvaginal ultrasonography in grayscale demonstrating a uterus measuring 6.28 × 4.46 × 3.39 cm (volume 49.72 cm³) with an empty uterine cavity and endometrial thickness of 3.95 mm, confirming the absence of an intrauterine gestational sac at the time of presentation.

Eight days after initial presentation, the patient underwent diagnostic laparoscopy with concurrent hysteroscopy and dilation and curettage (D&C) under general endotracheal anesthesia. Intraoperatively, the uterus measured 8-9 cm. A left interstitial ectopic pregnancy measuring 3-4 cm was identified, highly vascular, fused to the left fundus of the uterus with a broad base, and without evidence of rupture or hemoperitoneum (Figure 4). The endometrial cavity appeared clean on hysteroscopy. Endocervical and endometrial curettings were sent to pathology. Estimated blood loss was less than 5 mL.

**Figure 4:**
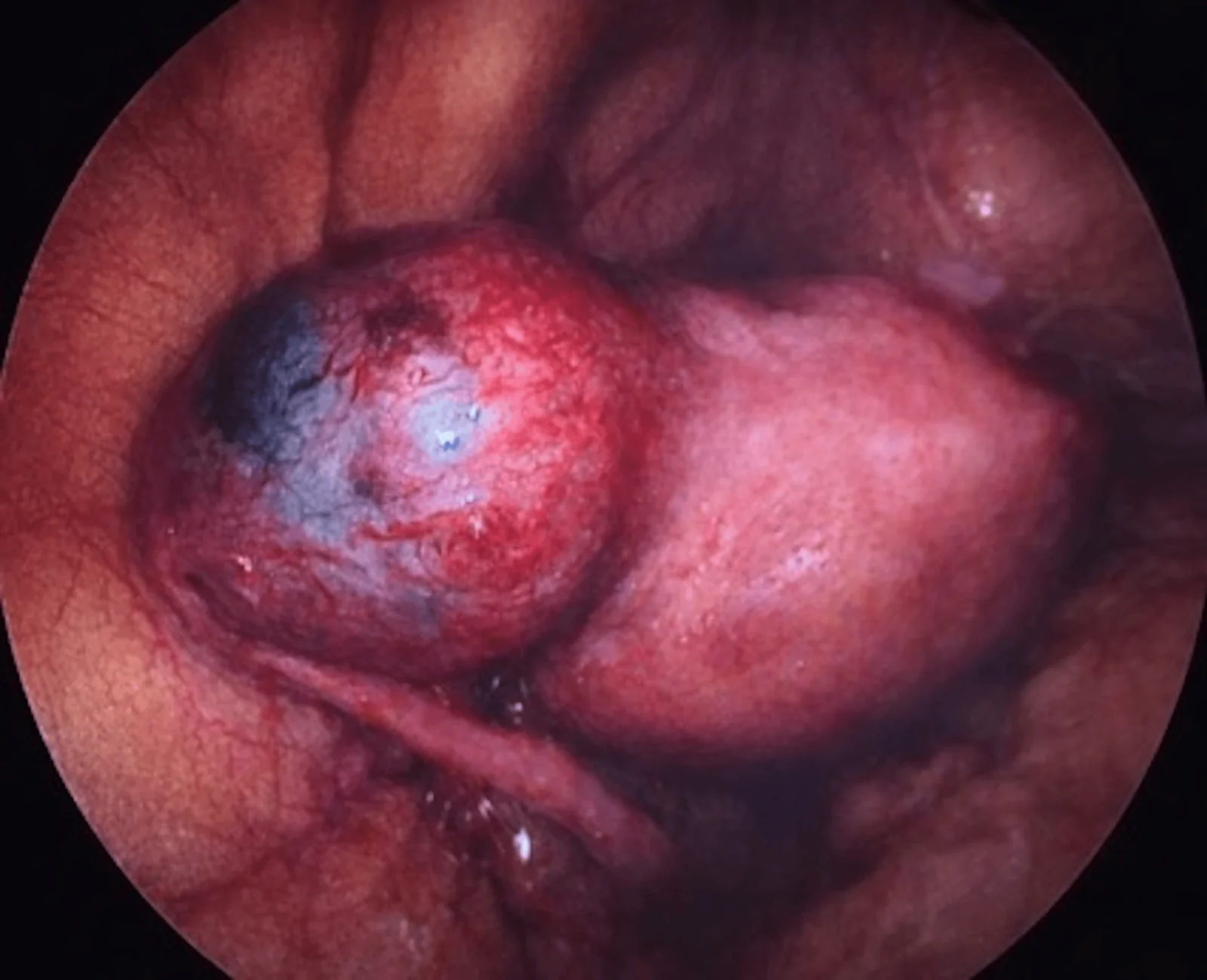
Intraoperative laparoscopic photograph demonstrating a 3–4 cm left interstitial ectopic pregnancy arising from the left interstitial region of the uterus. The mass is highly vascular with prominent surface vascularity and central bluish-purple discoloration consistent with trophoblastic tissue. The absence of rupture or hemoperitoneum confirmed hemodynamic stability and informed the intraoperative decision to pursue conservative management with systemic multi-dose methotrexate with leucovorin rescue.

Given the exceptional vascularity of the interstitial mass and the associated risk of catastrophic hemorrhage with immediate wedge resection, the decision was made intraoperatively to defer resection and pursue systemic multi-dose methotrexate with leucovorin rescue. The abdomen was desufflated, the port sites were closed, and the patient was transferred to the recovery room in stable condition.

Beginning the day after surgery, the patient received multi-dose systemic methotrexate (1 mg/kg intramuscularly on Days 1, 3, and 5) with leucovorin rescue (0.1 mg/kg intramuscularly on Days 2, 4, and 6), as per the multi-dose alternating protocol. Daily complete blood counts and hepatic function panels were monitored throughout the inpatient course. Serum β-hCG was trended daily. The patient tolerated the regimen without significant adverse effects, reporting only mild nausea. Her hemoglobin remained stable (12.5 g/dL by Day 3) and liver function tests remained stable without clinically significant elevation. The laparoscopic incision healed without complication. She was discharged on hospital day 7, with instructions for serial outpatient β-hCG surveillance until levels reached an undetectable threshold (< 5 mIU/mL). On subsequent outpatient follow-up, serial β-hCG surveillance demonstrated decline to an undetectable threshold (< 5 mIU/mL). However, interval imaging identified a persistent left adnexal mass and a newly developed left hydrosalpinx. Because residual masses following methotrexate may take weeks to months to resolve, the persistent mass alone was not considered an indication for surgery. The newly developed hydrosalpinx, however, warranted surgical management. Hydrosalpinx is associated with reduced spontaneous fertility and poorer outcomes with assisted reproduction, and salpingectomy improves in vitro fertilization success rates. Given the patient's desire for future fertility, she was counseled regarding left salpingectomy.

Approximately eight weeks after initial presentation, the patient underwent laparoscopic left salpingectomy with wedge resection of the left uterine cornu. The left fallopian tube was identified, and the mesosalpinx was divided using a LigaSure device (Medtronic, Minneapolis, MN, USA). Rather than transecting the tube near the uterine cornu, as in a conventional salpingectomy, a wedge resection of the left cornu was performed with the LigaSure device, allowing en bloc removal of the tube together with the residual interstitial mass and surrounding myometrium (Figures 5, 6; Appendices). The surgical field was irrigated and inspected for hemostasis, with electrocautery applied as needed. The hemostatic agents Surgiflo® and Surgicel® (Ethicon, Raritan, NJ, USA) were applied to the surgical site. The specimen was retrieved in a laparoscopic specimen bag and sent to pathology. All ports were removed under direct visualization, and port sites were closed in standard fashion. The patient was admitted overnight for postoperative observation.

**Figure 5:**
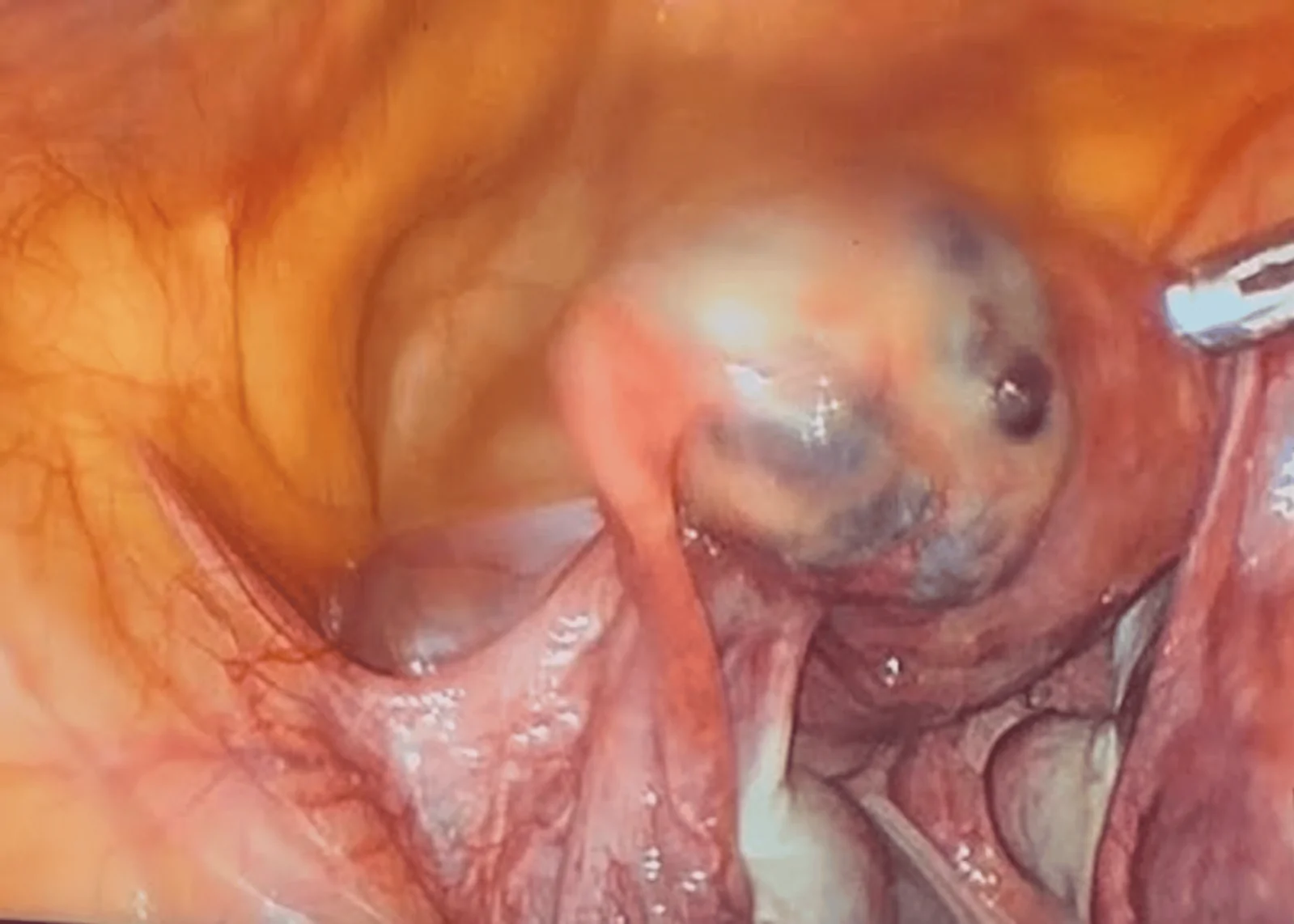
Intraoperative laparoscopic photograph demonstrating a 3–4 cm left interstitial ectopic pregnancy arising from the left interstitial region of the uterus and corresponding hydrosalpinx. When compared to the initial intraoperative diagnostic laparoscopic photograph, the mass is less vascularized as demonstrated by its white-ish hue. Absence of rupture or hemoperitoneum confirmed.

**Figure 6:**
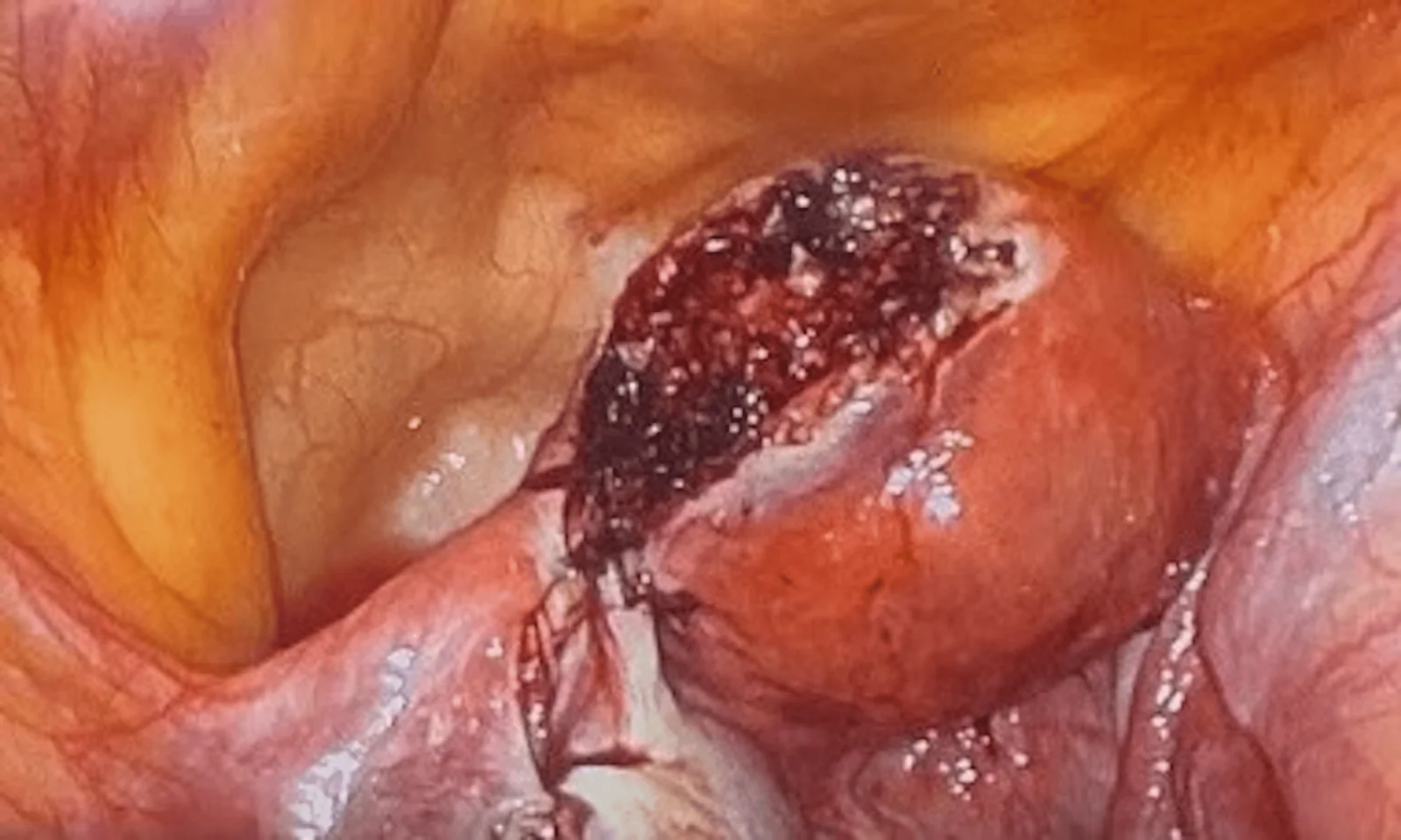
Intraoperative laparoscopic photograph demonstrating left salpingectomy with wedge resection.

The resected specimen consisted of a left fallopian tube measuring 10.0 cm in length and 4.5 cm in greatest diameter, with a dilated lumen (Figure 7). A firm nodular lesion measuring 4.5 × 4.5 × 3.0 cm was present at the proximal (interstitial) end of the tube. Its serosal surface was smooth and glistening, with nodular areas of hemorrhage, and sectioning revealed white-to-hemorrhagic tissue. No fetal tissue was grossly identified. Histopathologic examination of the lesion demonstrated an organizing thrombus with necrotic chorionic villi, consistent with the treated interstitial ectopic pregnancy.

**Figure 7:**
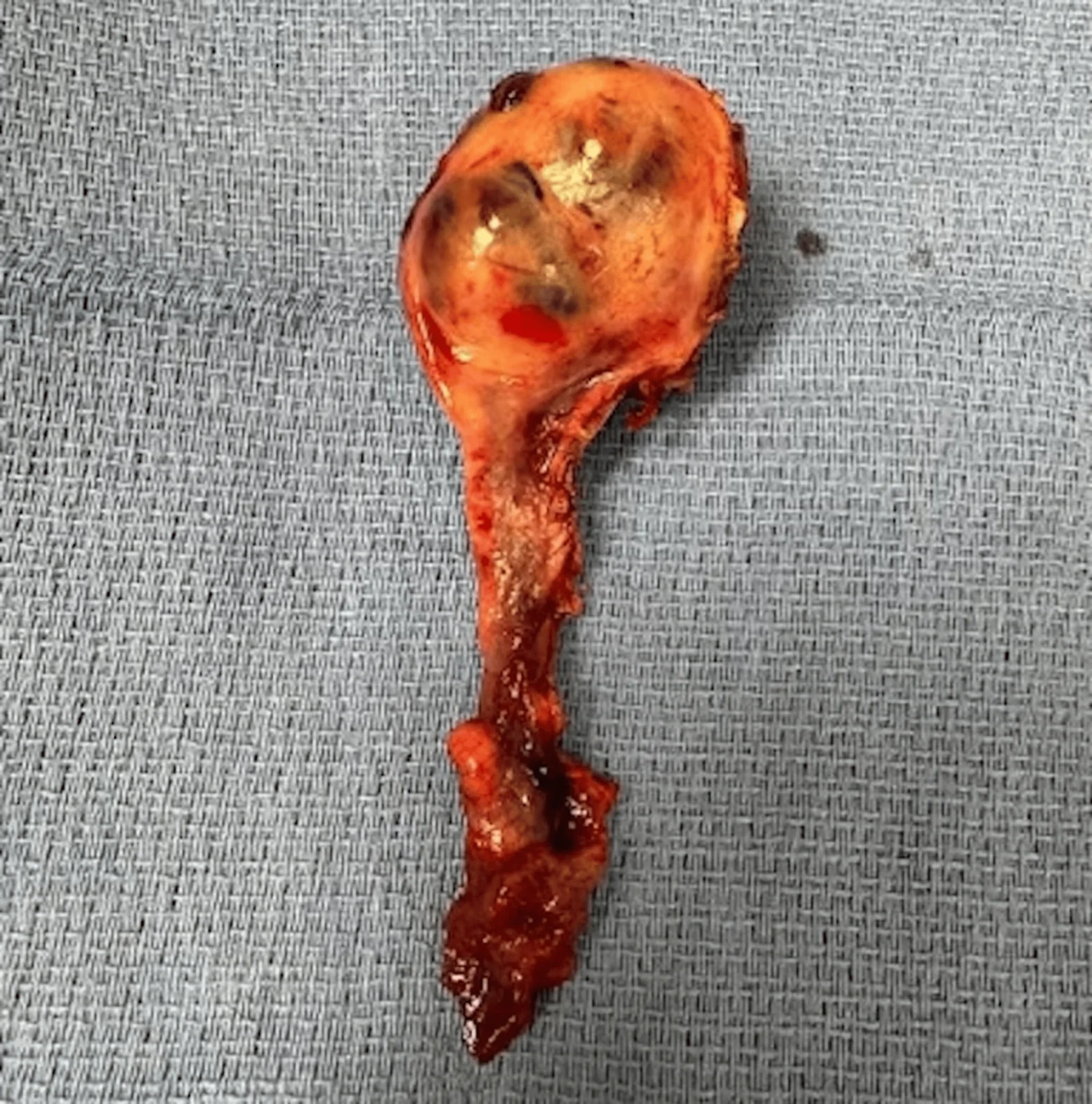
Postoperative photograph that depicts an approximately 3.0-3.5 cm left interstitial ectopic pregnancy with left fallopian tube and wedge resection specimen.

## Discussion

This case illustrates four clinically important dimensions of interstitial ectopic pregnancy management: the diagnostic limitations of TVUS in identifying an interstitial implantation site; the central role of serial β-hCG kinetics in evaluating a PUL; the feasibility of a uterus-conserving approach that combines diagnostic laparoscopy with postoperative systemic methotrexate; and the importance of long-term sonographic surveillance following medical management, given the risk of sequelae such as incomplete anatomical resolution with hydrosalpinx requiring subsequent surgical intervention, as occurred in this case.

Interstitial ectopic pregnancy is particularly hazardous because the surrounding myometrium affords greater structural support than the thin-walled fallopian tube, permitting growth to a larger size before rupture [3, 4]. When rupture does occur, hemorrhage from the interstitial vasculature, supplied by both the uterine and ovarian arteries, can be torrential and life-threatening. In this case, the interstitial mass measured 3-4 cm at the time of surgical diagnosis and remained unruptured, underscoring the value of early identification through serial clinical surveillance, even when ultrasonography is non-diagnostic. 

The TVUS performed at initial ED presentation was non-diagnostic for ectopic pregnancy, demonstrating no adnexal mass and no free fluid, findings that would typically be reassuring in most clinical contexts. However, three validated sonographic criteria for interstitial ectopic pregnancy have been described: an empty uterine cavity, a gestational sac located more than 1 cm from the lateral margin of the endometrial cavity, and a myometrial mantle of less than 5 mm surrounding the sac [5]. The 'interstitial line sign,' an echogenic line extending from the endometrial cavity to the interstitial sac, has a sensitivity of approximately 80% and specificity of 98% for interstitial ectopic pregnancy when present [4]. The absence of these findings before seven to eight weeks gestation is not uncommon, as the interstitial sac may be too small to resolve on ultrasound. This case reinforces that a negative or inconclusive TVUS does not exclude an ectopic pregnancy, particularly of the interstitial subtype, and that clinical suspicion must be maintained.

The serial β-hCG pattern in this patient was the pivotal diagnostic feature. Current evidence-based guidelines recommend that a minimum β-hCG rise of 53% over 48 hours is expected for a viable intrauterine pregnancy; values that plateau, rise suboptimally, or decline more slowly than expected are strongly associated with ectopic implantation and warrant further investigation [4]. In this patient, the β-hCG rose from 3,954 to 6,136 mIU/mL over five days, corresponding to an approximate 18.6% rise when normalized to a 48-hour interval, well below the expected minimum, then declined to 5,863 and subsequently 5,028 mIU/mL. This pattern of abnormal kinetics, in combination with an inconclusive TVUS and a clinically symptomatic presentation, appropriately triggered escalation to operative diagnosis.

The identification of a left corpus luteum on initial TVUS is a clinically relevant finding. The corpus luteum forms at the site of the ovulating follicle, and its ipsilateral location to the ultimately confirmed ectopic implantation is consistent with left-sided ovulation followed by aberrant tubal transport into the interstitial segment. As the corpus luteum is most commonly ipsilateral to the site of ectopic implantation, it serves as a useful lateralizing sign in the context of PUL [5].

Regarding management, the therapeutic decision in this case, to defer immediate wedge resection in favor of systemic methotrexate following diagnostic laparoscopy, reflects contemporary best practice for hemodynamically stable patients with unruptured interstitial ectopic pregnancies meeting pharmacologic eligibility criteria. Criteria for systemic methotrexate include hemodynamic stability, no evidence of rupture, patient compliance with follow-up, absence of contraindications (hepatic dysfunction, immunosuppression, renal insufficiency, and blood dyscrasias), and, for the multi-dose protocol, no cardiac activity on TVUS [3, 5]. Success rates for multi-dose methotrexate in interstitial ectopic pregnancy vary with initial β-hCG level, with reported success of approximately 80%-90% in published series [3]. The mildly elevated ALT noted in this patient (72 U/L) was taken into account and monitored closely; while transaminase elevation can be a relative contraindication at markedly elevated levels (typically greater than twice the upper limit of normal), the degree of elevation in this case was deemed clinically acceptable by the treating team in consultation with the Maternal-Fetal Medicine department.

Leucovorin (folinic acid) serves as a selective rescue agent in the multi-dose protocol, restoring tetrahydrofolate synthesis in normal host tissues, particularly the gastrointestinal mucosa, bone marrow, and hepatocytes, while permitting continued methotrexate-mediated inhibition of dihydrofolate reductase in trophoblastic tissue [7]. This pharmacologic selectivity allows for a higher cumulative methotrexate exposure relative to single-dose regimens, improving efficacy at the cost of greater monitoring requirements. The patient tolerated the full six-day protocol with only mild nausea, consistent with the expected side effect profile.

This case joins an expanding body of literature supporting nonsurgical or minimally invasive management of interstitial ectopic pregnancy in stable, compliant patients. The high vascularity and broad implantation base observed intraoperatively exemplify the clinical scenario in which immediate surgical excision carries substantial hemorrhagic risk, reinforcing the role of intraoperative judgment in safe care. However, this case also illustrates an important caveat: a declining β-hCG does not guarantee complete anatomical resolution. Despite achieving an undetectable β-hCG, this patient had a persistent left adnexal mass and developed a left hydrosalpinx, for which salpingectomy with wedge resection was performed to optimize future fertility. The implications for future pregnancy must therefore be weighed carefully. Wedge resection requires partial myometrial excision, which may compromise uterine integrity in subsequent pregnancies and necessitate cesarean delivery. Methotrexate, by contrast, avoids immediate uterine trauma, but as in the present case, incomplete resolution may still lead to subsequent myometrial resection [3, 4]. Both approaches in this case were uterus-conserving, preserving the potential for future pregnancy rather than guaranteeing fertility. This underscores that long-term sonographic surveillance, not β-hCG kinetics alone, is essential after medical management.

Future reproductive outcomes warrant specific consideration following interstitial wedge resection. Removal of the interstitial segment and surrounding myometrium leaves a potentially weakened area of the uterine wall, and uterine rupture at the resection site has been reported in subsequent pregnancies, including before the onset of labor [3, 4]. Patients should therefore be counseled to delay conception to allow adequate myometrial healing, to receive close antenatal surveillance in future pregnancies, and to plan for cesarean delivery before the onset of labor. Salpingectomy was favored in this case because hydrosalpinx is associated with reduced spontaneous fertility and poorer outcomes with assisted reproduction, and its removal improves in vitro fertilization success rates. Because the right fallopian tube remains intact, spontaneous conception remains possible, and assisted reproduction is available if needed. The patient was counseled regarding these risks and future pregnancy planning.

## Conclusions

Interstitial ectopic pregnancy is a rare but potentially fatal condition that may present as a pregnancy of unknown location when TVUS is nondiagnostic. Serial serum β-hCG kinetics, particularly a pattern of suboptimal rise followed by plateau and decline, remain the most reliable noninvasive tool for identifying a nonviable or ectopic gestation when ultrasonography is inconclusive. Diagnostic laparoscopy provides definitive identification and the opportunity for simultaneous therapeutic decision-making. In hemodynamically stable patients with unruptured interstitial ectopic pregnancies and high intraoperative hemorrhagic risk, systemic multi-dose methotrexate with leucovorin rescue is a safe, uterus-conserving initial approach. However, as this case demonstrates, β-hCG resolution does not assure complete anatomical regression, and the development of hydrosalpinx may warrant salpingectomy with wedge resection, particularly in patients desiring future fertility. Long-term sonographic surveillance following medical management is essential. Definitive treatment of interstitial pregnancy remains salpingectomy with wedge resection, particularly in cases of rupture, tubal damage, or incomplete resolution after conservative management, as in the present case.
